# Impact of type 2 diabetes mellitus on short- and long-term mortality after coronary artery bypass surgery

**DOI:** 10.1186/s12933-018-0796-7

**Published:** 2018-11-29

**Authors:** Alexander Kogan, Eilon Ram, Shany Levin, Enrique Z. Fisman, Alexander Tenenbaum, Ehud Raanani, Leonid Sternik

**Affiliations:** 1 Department of Cardiac Surgery, Sheba Medical Center, Tel Hashomer, Affiliated to the Sackler School of Medicine, Tel Aviv University, 52621 Tel Aviv, Israel; 2 Cardiac Surgery Intensive Care Unit, Sheba Medical Center, Tel Hashomer, Affiliated to the Sackler School of Medicine, Tel Aviv University, Tel Aviv, Israel; 3 Tel Aviv University, Tel Aviv, Israel

**Keywords:** Diabetes mellitus, Coronary artery bypass grafting, Revascularization, Insulin

## Abstract

**Background:**

Type 2 diabetes mellitus (DM) is a frequent co-morbidity among patients undergoing coronary artery bypass grafting (CABG) surgery. The aim of this study was to evaluate the impact of DM on the early- and long-term outcomes of patients who underwent isolated CABG.

**Methods:**

We performed an observational cohort study in a large tertiary medical center over a period of 11 years. All data from patients who had undergone isolated CABG surgery between 2004 and 2014 were obtained from our departmental database. The study population included 2766 patients who were divided into two groups: Group I (1553 non-diabetic patients), and Group II (1213 patients suffering from type 2 DM). Group II patients were then divided into two subgroups: subgroup IIA (981 patients treated with oral antihyperglycemic medications) and subgroup IIB (232 insulin-treated patients with or without additional oral antihyperglycemic drugs). In-hospital, 1-, 3-, 5- and 10-year mortality outcome variables were evaluated. Mean follow-up was 97 ± 41 months.

**Results:**

In-hospital mortality was similar between Group I and Group II patients (1.87% vs. 2.31%, p = 0.422) and between the subgroups IIA and IIB (2.14% vs. 3.02%, p = 0.464). Long-term mortality (1, 3, 5 and 10 years) was higher in Group II (DM type 2) compared with Group I (non-diabetic patients) (5.3% vs. 3.6%, p = 0.038; 9.3% vs. 5.6%, p < 0.001; 15.3% vs. 9.3%, p < 0.001 and 47.3% vs. 29.6% p < 0.001). Kaplan–Meier analysis demonstrated that all-cause mortality was higher in Group II compared with Group I (p < 0.001) and in subgroup IIB compared with subgroup IIA (p = 0.001). Multivariable analysis showed that DM increased the mortality hazard by twofold, and among diabetic patients, insulin treatment increased the mortality hazard by twofold.

**Conclusions:**

Diabetic and non-diabetic patients have similar in-hospital mortality rates. Survival rates of diabetic patients start to deteriorate 3 year after surgery. Type 2 DM is an independent predictor for long-term mortality after isolated CABG surgery. Mortality is even higher when the diabetes treatment strategy included insulin.

**Electronic supplementary material:**

The online version of this article (10.1186/s12933-018-0796-7) contains supplementary material, which is available to authorized users.

## Introduction

Diabetes mellitus (DM) doubles the risk of cardiovascular disease [1] and about 75% of deaths in diabetic patients are due to coronary artery disease [2]. Studies performed during the 1980’s and 90’s demonstrated increasing short- [3] and long-term mortality in diabetic patients undergoing CABG compared with non-diabetic patients [4, 5]. However, more recently, reports have shown a significant reduction in mortality among patients with diabetes [6]. The aim of the current study was to investigate the impact of DM type 2 on short- and long-term mortality. The study was conducted on a large cohort of patients undergoing CABG.

## Methods

The study was approved by the Sheba Medical Center Institutional Ethical Committee (Protocol No 4257). The requirement for informed consent was waived because of the retrospective nature of the study. We performed a retrospective, observational study that included prospectively-collected data from consecutive patients who had undergone a first isolated CABG at a large tertiary university hospital over an 11-year period, between 01.04.2004 and 31.03.2015. Patients were divided into two groups: Group I (non-diabetic patients), and Group II (patients suffering from type 2 DM). DM type 2 was defined according to the American Diabetes Association as: (a) hemoglobin A1C ≥ 6.5%; (b) fasting plasma glucose levels ≥ 126 mg/day; (c) classic symptoms of hyperglycemia or a hyperglycemic crisis, a random plasma glucose level ≥ 200 mg/dL (11.1 mmol/L) [7]; or (d) currently on pharmacologic treatment (oral antihyperglycemic drugs and/or insulin). Group II patients were divided into two subgroups: subgroup IIA, treated only with oral antihyperglycemics and subgroup IIB, treated with insulin or a combination of oral antihyperglycemic drugs and insulin.

Using non-identifiable patient data from our department’s database, we evaluated the following variables: gender, age, chronic obstructive pulmonary disease, congestive heart failure [New York Heart Association (NYHA) functional class], DM, dialysis-dependent renal failure, peripheral vascular disease, a previous cardiovascular accident (CVA)/transient ischemic attack (TIA), systemic and pulmonary hypertension, left ventricular function, priority of surgery (elective, urgent, or emergent), and logistic EuroSCORE I. Perioperative variables included using left and right internal mammary arteries, the number of anastomosis, time of cross-clamping and duration of cardiopulmonary bypass.

After surgery all patients were admitted to the intensive care unit (ICU) directly from the operating room. After discharge from the ICU, patients were transferred either to a step-down unit or directly to the floor, from where they were discharged either to their home or to a rehabilitation facility. In the operating room and in the ICU, patients from both groups received intravenous continuous infusion of regular insulin according to the Society of Thoracic Surgeons practice guideline series [8]. After discharge from the ICU, patients from Group I (non-diabetic) did not receive insulin or any other hypoglycemic medication, while patients from Group II continued to receive preoperative antiglycemic treatment (per oral drugs and insulin), as soon as they began to eat. We evaluated the following outcome variables: in-hospital, 1-, 3-, 5- and 10-year mortality. Mortality was ascertained from the Israeli Ministry of Interior Population Register. Mean follow-up was 98 ± 41 months and completed for all patients.

Throughout the study no major changes in hospital policy, surgical or anesthesiological techniques were introduced. All patients received standardized anesthesia.

### Database management and statistical analysis

At discharge, all patient data were checked, corrected and entered into a database. Descriptive statistics were used to summarize data, and numerical data were expressed as means (SD). Normality of the distribution of continuous data variables was analyzed using the Kolmogorov–Smirnov test. Since not all numerical data were distributed normally, Mann–Whitney U-tests were used to evaluate differences between groups. Differences between the frequencies of categorical variables were also estimated using Fisher’s exact test. All outcome variables were compared between whole groups of patients.

Variables that had a possible influence on patient mortality (univariate predictors) were evaluated using Fisher’s exact test. Suspected predictors for mortality by univariable analysis were analyzed by a multivariable logistic regression analysis in order to find independent predictors of mortality. The variables included in the final model among the entire cohort were: DM, Euroscore, NYHA functional class, and hyperlipidemia. The variables included in the final model among the diabetic patients were: insulin therapy, Euroscore, NYHA functional class, and hyperlipidemia. p values of 0.05 and less were considered as statistically significant. All statistical analyses were performed using SPSS 11.5 for Windows.

## Results

### Baseline characteristics

During the 11-year study period, 2972 patients underwent isolated CABG, of whom 36 patients were excluded from the study due to incomplete data, and a further 170 patients were excluded since they suffered from DM type 1. The study population included 2766 patients (Table 1). Group I (non-diabetic patients), comprised 1553 patients, and Group II (patients suffering from DM type 2), comprised 1213 patients. Of them, 981 patients were treated with oral antihyperglycemic medications (Group IIA) and 232 patients were treated with insulin (Group IIB) (Table 2). Compared with the non-diabetic group, the diabetic group of patients were older, more frequently women, had a higher mean logistic EuroSCORE, and had a higher prevalence of systemic and pulmonary hypertension, peripheral vascular disease and previous CVA.

**Table 1:** Patients’ data (Groups I and II)

	Non-DM Group I No. of patients (1553)	DM Group II No. of patients (1213)	p value
Age (years)	63 ± 11	65 ± 10	0.000
Males	1308 (84%)	947 (78%)	0.000
Elective	978 (63%)	788 (65%)	0.378
NYHA FC III–IV	373 (20%)	376 (27%)	0.001
Previous operation	32 (2%)	37 (3%)	0.110
Ejection fraction (%)	51 ± 11	50 ± 11	0.001
Logistic EuroSCORE	5.6 ± 8.2	6.4 ± 9.6	0.020
Hypertension	1065 (69%)	1052 (87%)	0.000
COPD	70 (4%)	61 (5%)	0.529
Dialysis	17 (1%)	24 (2%)	0.059
Hyperlipidemia	1097 (71%)	1019 (84%)	0.000
PVD	111 (7%)	185 (15%)	0.000
CVA/TIA	100 (6%)	130 (11%)	0.000
Pulmonary hypertension	3 (0.2%)	16 (1%)	0.001
Arrhythmia	8 (0.5%)	6 (0.5%)	1.000
LIMA	1509 (97%)	1171 (97%)	0.378
No mammary	26 (2%)	28 (2%)	1.000
Double mammary	781 (50%)	468 (39%)	0.000
Number of grafts	3.1 ± 1.1	3.1 ± 1.0	0.411
Bypass	80 ± 27	82 ± 42	0.133
Clamp	56 ± 20	56 ± 29	0.701
Mini invasive	33 (2%)	21 (2%)	0.491

*DM* Diabetes mellitus, *NYHA FC* New York Heart Association functional class, *COPD* chronic obstruction pulmonary disease, *PVD* peripheral vascular disease, *CVA* cerebral vascular accident, *TIA* transient ischemic attack, *LIMA* left internal mammary artery

**Table 2:** Patients’ data (subgroups IIA and IIB)

	Non-insulin No. of patients = 981	Insulin No. of patients = 232	p value
Age (years)	65 ± 10	64 ± 10	0.023
Males	775 (79%)	172 (74%)	0.113
Elective	627 (65%)	149 (65%)	1.000
NYHA FC III–IV	284 (26%)	88 (31%)	0.003
Previous operation	28 (3%)	9 (4%)	0.399
Ejection fraction (%)	51 ± 11	47 ± 12	0.000
Logistic EuroSCORE	6 ± 9	8 ± 11	0.012
Hypertension	847 (86%)	205 (88%)	0.453
COPD	51 (5%)	10 (4%)	0.738
Dialysis	12 (1%)	12 (5%)	0.001
Hyperlipidemia	818 (83%)	201 (87%)	0.273
PVD	125 (13%)	60 (26%)	0.000
CVA/TIA	106 (11%)	24 (10%)	0.906
Pulmonary hypertension	10 (1%)	6 (3%)	0.099
Arrhythmia	2 (0.2%)	4 (2%)	0.014
LIMA	949 (97%)	222 (96%)	0.426
Double mammary	396 (40%)	72 (31%)	0.032
Number of grafts	3 ± 1	3 ± 1	0.231
Bypass	83 ± 42	81 ± 40	0.471
Clamp	57 ± 31	53 ± 17	0.014
Mini invasive	18 (2%)	3 (1%)	0.781

*NYHA FC* New York Heart Association functional class, *COPD* chronic obstruction pulmonary disease, *PVD* peripheral vascular disease, *CVA* cerebral vascular accident, *TIA* transient ischemic attack, *LIMA* left internal mammary artery

### Early outcomes

Overall in-hospital mortality was similar between Groups I and II: 1.87% vs. 2.31%, p = 0.422, and between subgroups IIA and IIB: 2.14% vs. 3.01%, p = 0.464. Other in-hospital major events were similar between Groups I and II, such as CVA (0.3% vs. 0.5%, p = 0.350), use of intra-aortic balloon pump (5% vs. 5%, p = 0.859) and renal replacement therapy (0.7% vs. 1.5%, p = 0.059). Major events were also similar between the non-insulin dependent and insulin-dependent DM patients: CVA (0.5% vs. 0.4%, p = 1.000), use of intra-aortic balloon pump (5% vs. 6%, p = 0.239) and renal replacement therapy (1% vs. 2%, p = 0.364). Group II compared to Group I, and Group IIB compared to Group IIA experienced a significantly higher rate of prolonged mechanical ventilation time (over 72 h) (5.6% vs. 3.1%, p = 0.001 and 9.7% vs. 4.7%, p = 0.006), > 7 days in the ICU (5.7% vs. 2.7%, p < 0.001 and 9.7% vs. 4.8%, p = 0.007) and a significantly longer hospital duration (7.6 ± 7.9 vs. 6.7 ± 5.2, p < 0.001 and 9 ± 8 vs. 7 ± 8, p = 0.042).

### Follow-up mortality

**Fig. 1:**
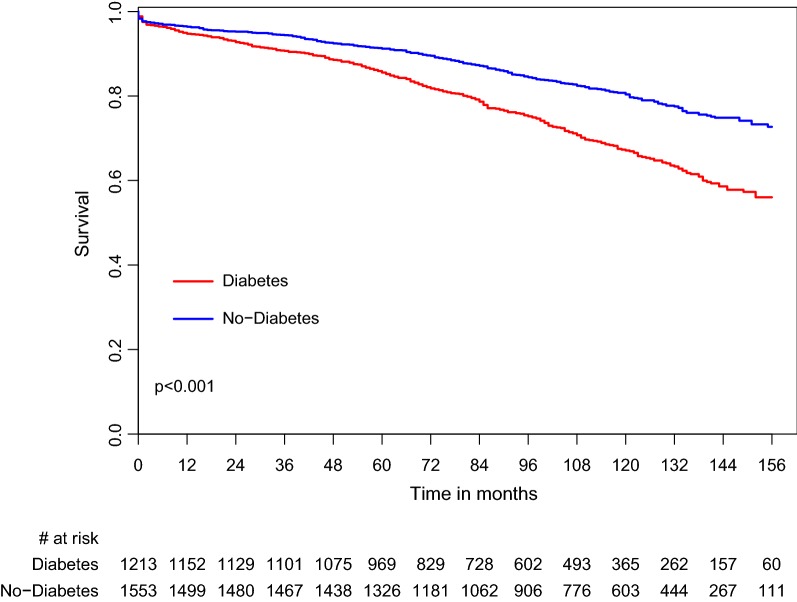
Survival rate by DM groups. *DM* Diabetes mellitus

Long-term mortality (1, 3, 5 and 10 years) was higher in Group II (DM type 2) compared with Group I (non-diabetic patients) (5.3% vs. 3.6%, p = 0.038; 9.3% vs. 5.6%, p < 0.001; 15.3% vs. 9.3%, p < 0.001 and 47.3% vs. 29.6%, p < 0.001) (Fig. 1; Additional file 1: Table S1). These results were also consistent among the subgroup of patients with NYHA functional class I–II and III–IV (Additional file 2: Figure S1 and Additional file 3: Figure S2).

**Fig. 2:**
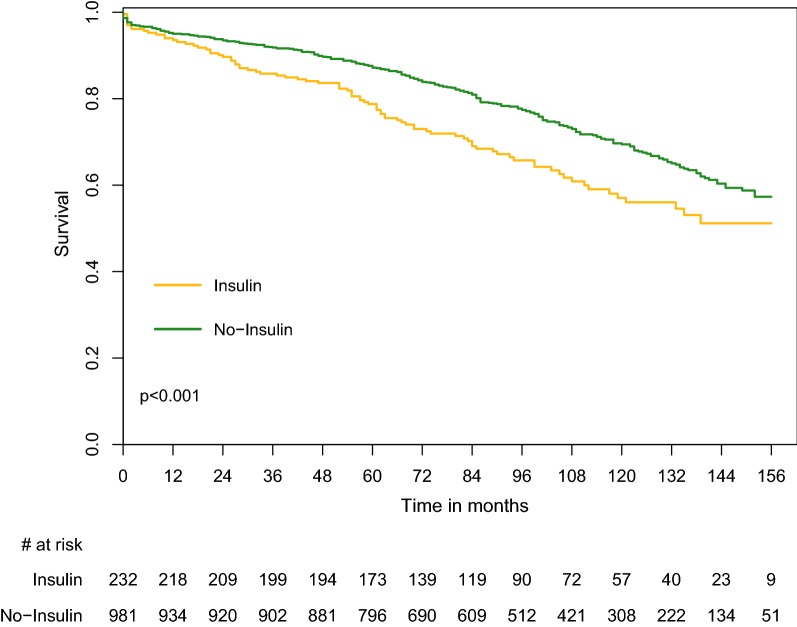
Survival rate in the DM group by insulin treatment. *DM* Diabetes mellitus

Furthermore, long-term mortality was higher in subgroup IIB (insulin-treated patients) compared to subgroup IIA (non-insulin treated patients) with 1-, 3-, 5- and 10-year mortality rates of 6.5% vs. 5%, p = 0.413; 14.2% vs. 8.2%, p = 0.008; 22.1% vs. 13.6%, p = 0.002 and 59.3% vs. 44.3%, p = 0.002 (Fig. 2; Additional file 1: Table S2). These results were also consistent among the subgroup of patients with NYHA functional class I–II and III–IV (Additional file 4: Figure S3 and Additional file 5: Figure S4).

Multivariable analysis demonstrated that predictors for 10 years’ mortality were higher on EuroSCORE (HR 1.38 95% CI 1.31–1.46, p < 0.001), hyperlipidemia (HR 1.39 95% CI 1.02–1.92, p = 0.037) and DM (HR 2.08 95% CI 1.57–2.74, p < 0.001). Among the diabetic patients, predictors for 10 years’ mortality were higher on EuroSCORE (HR 1.37 95% CI 1.3–1.45, p < 0.001), hyperlipidemia (HR 1.45 95% CI 1.06–1.97, p = 0.021) and insulin treatment (HR 2.11 95% CI 1.34–3.3, p = 0.001). Although higher NYHA functional class was a risk factor for late mortality by univariable analysis (p = 0.001), it was not associated with late mortality after adjustment for confounders by multivariable analysis, both in the entire cohort (p = 0.834) and among the diabetic patients (p = 0.831).

## Discussion

Our study investigated the impact of DM type 2 on in-hospital and long-term mortality in patients after their first isolated CABG. First, we found that among patients undergoing CABG, diabetic and non-diabetic patients had similar in-hospital mortality. Second, our principal finding was that the long-term mortality of diabetic patients was higher than that of non-diabetic patients. Furthermore, mortality was even higher when the diabetic treatment strategy included insulin compared to treatment without insulin. Macrovascular disease (coronary artery disease, stroke, and peripheral vascular disease) was responsible for the majority of morbidity and mortality associated with type 2 DM [9]. It should be pointed out that incident diabetes in adults is associated with a substantial risk for mortality, especially in younger adults [10], and in the presence of coronary artery disease (CAD) early revascularization is overwhelmingly important, since a deferred revascularization procedure in patients with and without DM shows that the former are associated with a significantly higher target lesion failure rate [11].

The impact of diabetes on short-term mortality after CABG was insignificant. Abizaid et al. [12] reported similar in-hospital and 1-year mortality rates for diabetic and non-diabetic patients: 2.1% vs. 1.2% and 3.1% vs. 2.8%. In addition, Marui et al. [13] found no differences in 30-day and 1-year mortality between diabetic and non-diabetic patients: 0.9% vs. 1.2% and 4.4% vs. 4.5%. Carson et al. [3] reported a 30-day mortality rate of 3.7% in patients with DM and 2.7% in those without DM. While Li et al. [14] reported similar post-CABG mortality rates for non-diabetic and diabetic patients treated with oral antihyperglycemic medication (1.88% vs. 2.01%), they showed a significant increase in mortality in a subgroup of diabetic patients treated with insulin (3.08%). Zalewska-Adamiec et al. [15] found no differences in 30-day, 1- and 2-year mortality rates (11.6% vs. 11.1%) in diabetic and non-diabetic patients with left main coronary artery disease. Whang et al. [16] found no differences in 2-year mortality rates between diabetic and non-diabetic patients: 26% vs. 24%, and concluded that diabetes was not a predictor of mortality after CABG among patients with left ventricular dysfunction. Although diabetic patients in our series were older and had more comorbidities, differences in early mortality did not reach statistical significance. We report here that in-hospital mortality among diabetic (N = 1553) and non- diabetic patients (N = 1213), was 2.3% and 1.9%. With these rates of mortality in a group the size of ours, we achieved a power of 13% to detect differences between the groups. Even if we would have reached the statistical significance, the clinical implications of these differences would have been questionable, since it is obvious that the most important impact of DM is on long-term results. In the non-insulin compared with the insulin-treated subgroup of patients, mortality was 2.14% vs. 3.01%, p = 0.464, and at 1 year, mortality was 5% vs. 6.5%, p = 0.413. In this context, it should be stressed that among hospitalized heart failure patients with no pre-existing DM there is a linear relationship between admission blood glucose and long-term mortality, whereas among patients with DM only an admission level of > 200 mg/dL is associated with increased mortality risk [17].

However, the impact of diabetes on long-term mortality after CABG has been controversial. Marui et al. [13] reported an increase in 3- and 5-years’ mortality in diabetic compared to non-diabetic patients, 11% vs. 9.7% and 19.6% vs. 16.2%. Mohamadi et al. [18] investigated only cardiac-specific survival rates, but not all-cause mortality, and postulated that DM type 2 diabetes is not an independent predictor of late cardiac death 6 years after CABG. Furthermore, Onuma et al. [19] reported slightly increasing mortality rates in diabetic compared with non-diabetic patients 5 years after CABG: 8.6% vs. 7.1%. Kappetein et al. [20] also investigated 5 years’ mortality after CABG and reported non-significant differences between diabetic and non-diabetic patients: 12.9% vs. 10.9%. However, Koshizaka et al. [21] reported significant differences in 5 years’ mortality: 15.5% vs. 8.5%. Wit et al. [22] reported significantly higher 3-year mortality rates in patients with insulin-treated compared with non-insulin-treated DM (16.7% vs. 8.7%) and non-diabetic patients (6.3%). In their systematic reviews and meta-analyses Bundhun et al. [23] postulated that diabetes is associated with increased long-term mortality after CABG, while CABG was associated with significantly lower long-term adverse clinical outcomes compared to percutaneous coronary intervention (PCI) in patients with insulin-treated type 2 DM [24].

While in the general population DM is associated with excess mortality, compared with the general population without DM, with a hazard ratio of 1.15 at 5-years [25], we reported a greater impact of DM on patients who underwent CABG (HR of 1.68 at 5 years). Although we have no data regarding the cause of death, myocardial infarction, and graft patency during the follow-up period, we assume that this higher impact of DM in our cohort, compared to the natural history of DM in the general population, is due to accelerated CAD, based on previous studies that support this theory [26].

In our report the 5-year mortality rate was 15.3% among diabetic patients and 9.3% among non-diabetic patients. Although we did not compare our results to a percutaneous approach, based on previous reports among diabetic and non-diabetic patients with multi-vessel CAD who were treated by PCI, our results were somewhat better. Among diabetic patients, Farkouh et al. [27] reported 5-year mortality rates of 26.6% in the PCI group. Contini et al. [28] reported a 5-year mortality rate of 24.5% in diabetic patients who underwent PCI. Kappetein et al. [20], throughout a 5-year follow-up, showed that PCI revealed a mortality rate of 19.5% in diabetic patients, and 12.0% in non-diabetic patients.

Only 18.5% of the patients in the current study were female, as reported in previous studies where the patients were also predominantly male [13, 18, 19]. The explanation for this observation could be that cardiovascular disease develops in an older age in females than in males, and older age increases the surgical risk. The differences in clinical presentation in women lead to less aggressive treatment strategies with less referral for surgical revascularization [29].

We reported that 1213 out of the 2766 patients (44%) who underwent isolated CABG in our Institute had diabetes, more than the average in previous reports [18–20]. Blumenfeld et al. [30] reported that in Israel, the threshold of patient referral for surgical revascularization has risen in the last two decades, and that those patients have more comorbidities than in the past. Since the advantage of CABG over PCI is seen mostly in patients with complex CAD, such as left-main stenosis or high SYNTAX score, and in diabetic patients, the characteristics of patients who are referred to CABG has changed during the last decade toward patients with a more complex anatomy and more diabetes. Recent reports based on National Registries have shown that 43–49% of the patients who were referred to CABG in Israel had diabetes [31, 32]. The current study is based on the population of a tertial referral center in Israel, and thus our report is consistent with real-life situations in Israel.

Diabetes mellitus is a chief cause of heart failure, either secondary to CAD or secondary to diabetic cardiomyopathy [33]. In general, insulin-dependent diabetic patients have more comorbidities than non-insulin dependent diabetic patients. Although the presence of insulin treatment is indeed a marker for more advanced disease, its underlying biological mechanism has not been fully elucidated. It may be related to the impact of a procoagulant imbalance, chronic exposure to high glucose levels, and direct effects of hyperinsulinemia. Interestingly, endogenous hyperinsulinemia has been associated with increased long-term mortality following myocardial infarction in patients without diabetes [34]. Further studies are needed to examine whether insulin-dependent diabetic patients should be included in risk stratification algorithms for patients who undergo CABG.

### Limitations

There are a few limitations in our study. First, while it is retrospective in design, data were collected prospectively and recorded in a well-defined database. Second, our study was conducted in a single-center cardiac surgery department. Third, we had no information regarding the main cause of death, the rate of cardiac events and data regarding graft patency during the follow-up period. Analysis of cardiac events could reinforce the conclusion that DM provides less favorable results after CABG. The lack of information regarding the main cause of death weakens the conclusions of this study.

## Conclusions

DM type-2 is an independent predictor of long-term mortality after CABG surgery. Mortality rates increased significantly when the diabetic treatment strategy included insulin. The high-risk population of insulin-dependent DM may require specific and/or more intense cardiovascular protective therapies after CABG. Further studies are needed to examine whether novel interventions, such as GLP-1 analogues or SGLT2 inhibitors, can improve their long-term outcomes. Since the current study is underpowered to detect reduced early survival in diabetic patients a larger study is warranted in order to reach significant differences in early mortality, which would enable us to conclude that DM may impact early results after CABG.

## Additional files

**Additional file 1: Table S1.** Life table for mortality in the entire cohort by patients with and without diabetes mellitus. **A**. Patients with diabetes mellitus. **B.** Patients without diabetes mellitus. **Table S2.** Life table for mortality among diabetic patients on insulin. **A.** Patients treated by insulin. **B**. Patients without insulin treatment.

**Additional file 2: Figure S1.** Survival rate by DM groups among patients with NYHA functional class I–II. DM = Diabetes mellitus; NYHA = New-York Heart Association.

**Additional file 3: Figure S2.** Survival rate by DM groups among patients with NYHA functional class III–IV. DM = Diabetes mellitus; NYHA = New-York Heart Association.

**Additional file 4: Figure S3.** Survival rate in the DM group receiving insulin treatment among patients with NYHA functional class I–II. DM = Diabetes mellitus; NYHA = New-York Heart Association.

**Additional file 5: Figure S4.** Survival rate in the DM group receiving insulin treatment among patients with NYHA functional class III–IV. DM = Diabetes mellitus; NYHA = New-York Heart Association.

## Abbreviations

CABG: coronary artery by-pass graft
CAD: coronary artery disease
CVA: cerebrovascular accident
DM: diabetes mellitus
ICU: intensive care unit
PCI: percutaneous coronary intervention
TIA: transient ischemic attack
