# Supplementary material for: Impact of type 2 diabetes mellitus on short- and long-term mortality after coronary artery bypass surgery
Source: Cardiovasc Diabetol. 2018 Nov 29;17:151. doi: 10.1186/s12933-018-0796-7 (PMC6264047; doi:10.1186/s12933-018-0796-7)
Supplement: Supplementary file 1 — Additional file 1: Table S1. Life table for mortality in the entire cohort by patients with and without diabetes mellitus. A. Patients with diabetes mellitus. B. Patients without diabetes mellitus. Table S2. Life table for mortality among diabetic patients on insulin. A. Patients treated by insulin. B. Patients without insulin treatment. [file 12933_2018_796_MOESM1_ESM.docx]

**Additional file 1**

**Table S1:** Life table for mortality in the entire cohort by patients with and without diabetes mellitus

1. Patients with diabetes mellitus

| **Time** | **Number at risk** | **Number of events** | **Survival rate** | **SE** | **95% CI** |
| --- | --- | --- | --- | --- | --- |
| 0 | 1213 | 14 | 0.988 | 0.00307 | 0.982-0.994 |
| 12 | 1152 | 50 | 0.947 | 0.00642 | 0.935-0.960 |
| 24 | 1129 | 24 | 0.927 | 0.00745 | 0.913-0.942 |
| 36 | 1101 | 25 | 0.907 | 0.00835 | 0.891-0.923 |
| 48 | 1075 | 26 | 0.885 | 0.00915 | 0.868-0.904 |
| 60 | 969 | 35 | 0.856 | 0.01014 | 0.836-0.876 |
| 72 | 829 | 40 | 0.818 | 0.01131 | 0.796-0.840 |
| 84 | 728 | 30 | 0.787 | 0.01224 | 0.763-0.811 |
| 96 | 602 | 30 | 0.752 | 0.01324 | 0.726-0.778 |
| 108 | 493 | 33 | 0.707 | 0.01457 | 0.679-0.736 |
| 120 | 365 | 22 | 0.671 | 0.01573 | 0.641-0.703 |
| 132 | 262 | 18 | 0.633 | 0.01723 | 0.600-0.668 |
| 144 | 157 | 16 | 0.586 | 0.01964 | 0.549-0.626 |
| 156 | 60 | 5 | 0.560 | 0.022 | 0.519-0.605 |

SE = Standard error; CI = Confidence interval.

1. Patients without diabetes mellitus

| **Time** | **Number at risk** | **Number of events** | **Survival rate** | **SE** | **95% CI** |
| --- | --- | --- | --- | --- | --- |
| 0 | 1553 | 26 | 0.983 | 0.00326 | 0.977-0.990 |
| 12 | 1499 | 30 | 0.964 | 0.00473 | 0.955-0.973 |
| 24 | 1480 | 18 | 0.952 | 0.00541 | 0.942-0.963 |
| 36 | 1467 | 13 | 0.944 | 0.00584 | 0.933-0.955 |
| 48 | 1438 | 30 | 0.925 | 0.0067 | 0.912-0.938 |
| 60 | 1326 | 19 | 0.912 | 0.0072 | 0.898-0.926 |
| 72 | 1181 | 23 | 0.895 | 0.00787 | 0.880-0.911 |
| 84 | 1062 | 30 | 0.872 | 0.00878 | 0.855-0.889 |
| 96 | 906 | 31 | 0.844 | 0.00977 | 0.825-0.864 |
| 108 | 776 | 20 | 0.824 | 0.01053 | 0.804-0.845 |
| 120 | 603 | 17 | 0.804 | 0.01139 | 0.782-0.826 |
| 132 | 444 | 19 | 0.775 | 0.01273 | 0.751-0.801 |
| 144 | 267 | 13 | 0.749 | 0.01432 | 0.721-0.777 |
| 156 | 111 | 5 | 0.727 | 0.01693 | 0.695-0.761 |

SE = Standard error; CI = Confidence interval.

**Table S2:** Life table for mortality among diabetic patients on insulin

1. Patients treated by insulin

| **Time** | **Number at risk** | **Number of events** | **Survival rate** | **SE** | **95% CI** |
| --- | --- | --- | --- | --- | --- |
| 0 | 232 | 1 | 0.996 | 0.0043 | 0.987-1.000 |
| 12 | 218 | 14 | 0.935 | 0.0161 | 0.904-0.968 |
| 24 | 209 | 9 | 0.897 | 0.02 | 0.858-0.937 |
| 36 | 199 | 9 | 0.858 | 0.0229 | 0.814-0.904 |
| 48 | 194 | 5 | 0.836 | 0.0243 | 0.790-0.885 |
| 60 | 173 | 11 | 0.788 | 0.027 | 0.736-0.842 |
| 72 | 139 | 12 | 0.730 | 0.0297 | 0.674-0.790 |
| 84 | 119 | 7 | 0.690 | 0.0317 | 0.631-0.755 |
| 96 | 90 | 5 | 0.657 | 0.0334 | 0.595-0.726 |
| 108 | 72 | 6 | 0.609 | 0.0364 | 0.541-0.684 |
| 120 | 57 | 4 | 0.570 | 0.0388 | 0.499-0.652 |
| 132 | 40 | 1 | 0.560 | 0.0394 | 0.488-0.643 |
| 144 | 23 | 3 | 0.512 | 0.045 | 0.431-0.608 |
| 156 | 9 | 0 | 0.512 | 0.045 | 0.431-0.608 |

SE = Standard error; CI = Confidence interval.

1. Patients without insulin treatment

| **Time** | **Number at risk** | **Number of events** | **Survival rate** | **SE** | **95% CI** |
| --- | --- | --- | --- | --- | --- |
| 0 | 981 | 13 | 0.987 | 0.00365 | 0.980-0.994 |
| 12 | 934 | 36 | 0.950 | 0.00696 | 0.937-0.964 |
| 24 | 920 | 15 | 0.935 | 0.00788 | 0.919-0.950 |
| 36 | 902 | 16 | 0.918 | 0.00874 | 0.901-0.936 |
| 48 | 881 | 21 | 0.897 | 0.0097 | 0.878-0.916 |
| 60 | 796 | 24 | 0.872 | 0.01072 | 0.851-0.893 |
| 72 | 690 | 28 | 0.839 | 0.01199 | 0.816-0.863 |
| 84 | 609 | 23 | 0.809 | 0.01305 | 0.784-0.835 |
| 96 | 512 | 25 | 0.774 | 0.01426 | 0.747-0.803 |
| 108 | 421 | 27 | 0.730 | 0.01578 | 0.700-0.762 |
| 120 | 308 | 18 | 0.694 | 0.01711 | 0.662-0.729 |
| 132 | 222 | 17 | 0.650 | 0.0191 | 0.614-0.689 |
| 144 | 134 | 13 | 0.603 | 0.02178 | 0.562-0.648 |
| 156 | 51 | 5 | 0.573 | 0.02471 | 0.527-0.623 |

SE = Standard error; CI = Confidence interval.
