# Supplementary figures and images for: Impact of type 2 diabetes mellitus on short- and long-term mortality after coronary artery bypass surgery
Source: Cardiovasc Diabetol. 2018 Nov 29;17:151. doi: 10.1186/s12933-018-0796-7 (PMC6264047; doi:10.1186/s12933-018-0796-7)

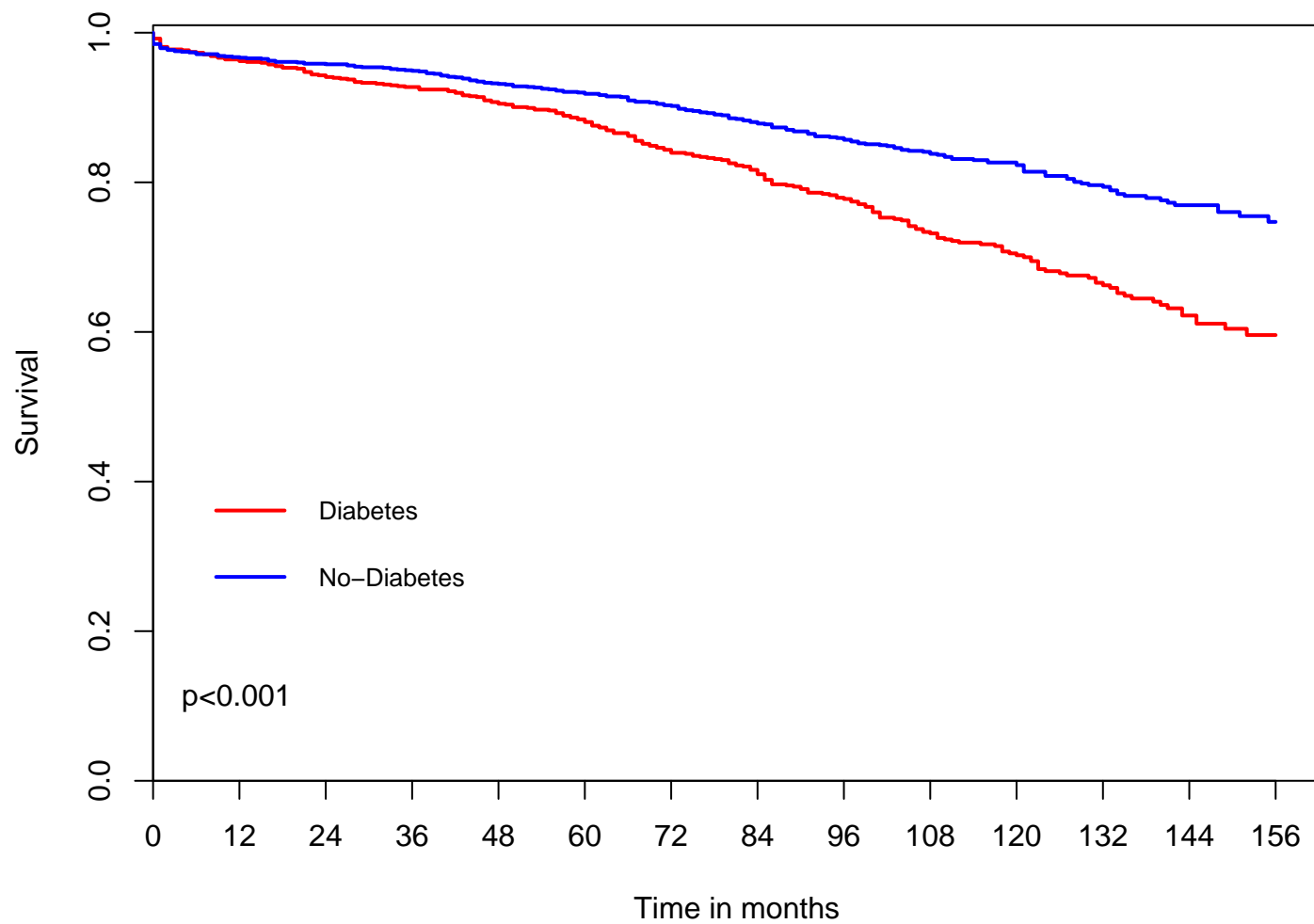

# at risk

|             | 0    | 12   | 24   | 36   | 48   | 60   | 72  | 84  | 96  | 108 | 120 | 132 | 144 | 156 |
|-------------|------|------|------|------|------|------|-----|-----|-----|-----|-----|-----|-----|-----|
| Diabetes    | 895  | 863  | 844  | 830  | 811  | 730  | 624 | 553 | 457 | 374 | 279 | 199 | 124 | 48  |
| No-Diabetes | 1255 | 1214 | 1203 | 1192 | 1170 | 1073 | 963 | 868 | 742 | 630 | 477 | 348 | 213 | 84  |

Supplement: Supplementary file 2 — Additional file 2: Figure S1. Survival rate by DM groups among patients with NYHA functional class I–II. DM = Diabetes mellitus; NYHA = New-York Heart Association. [file 12933_2018_796_MOESM2_ESM.pdf]

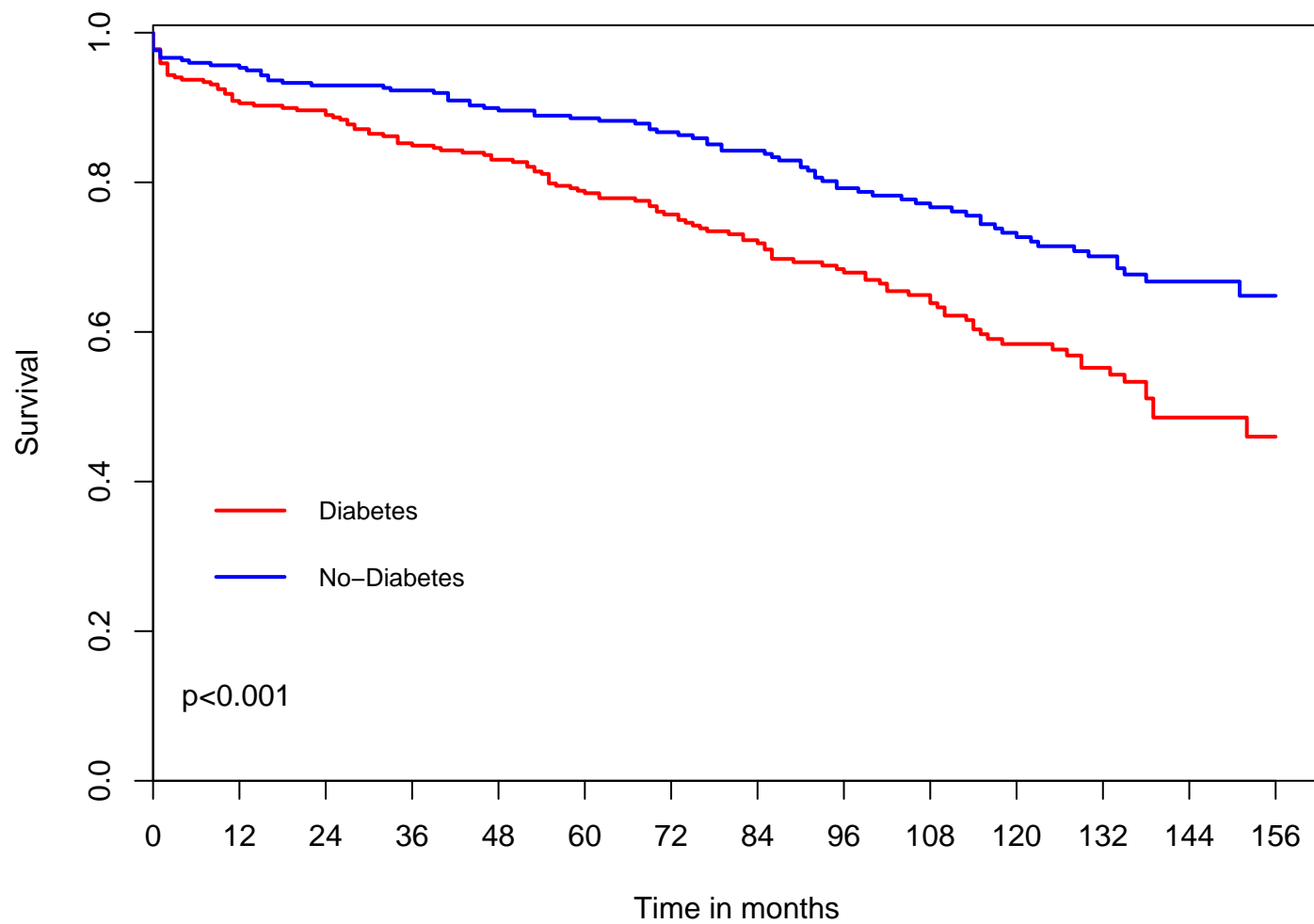

| # at risk   |  |     |     |     |     |     |     |     |     |     |     |     |    |    |    |
|-------------|--|-----|-----|-----|-----|-----|-----|-----|-----|-----|-----|-----|----|----|----|
| Diabetes    |  | 318 | 289 | 285 | 271 | 264 | 239 | 205 | 175 | 145 | 119 | 86  | 63 | 33 | 12 |
| No-Diabetes |  | 298 | 285 | 277 | 275 | 268 | 253 | 218 | 194 | 164 | 146 | 126 | 96 | 54 | 27 |

Supplement: Supplementary file 3 — Additional file 3: Figure S2. Survival rate by DM groups among patients with NYHA functional class III–IV. DM = Diabetes mellitus; NYHA = New-York Heart Association. [file 12933_2018_796_MOESM3_ESM.pdf]

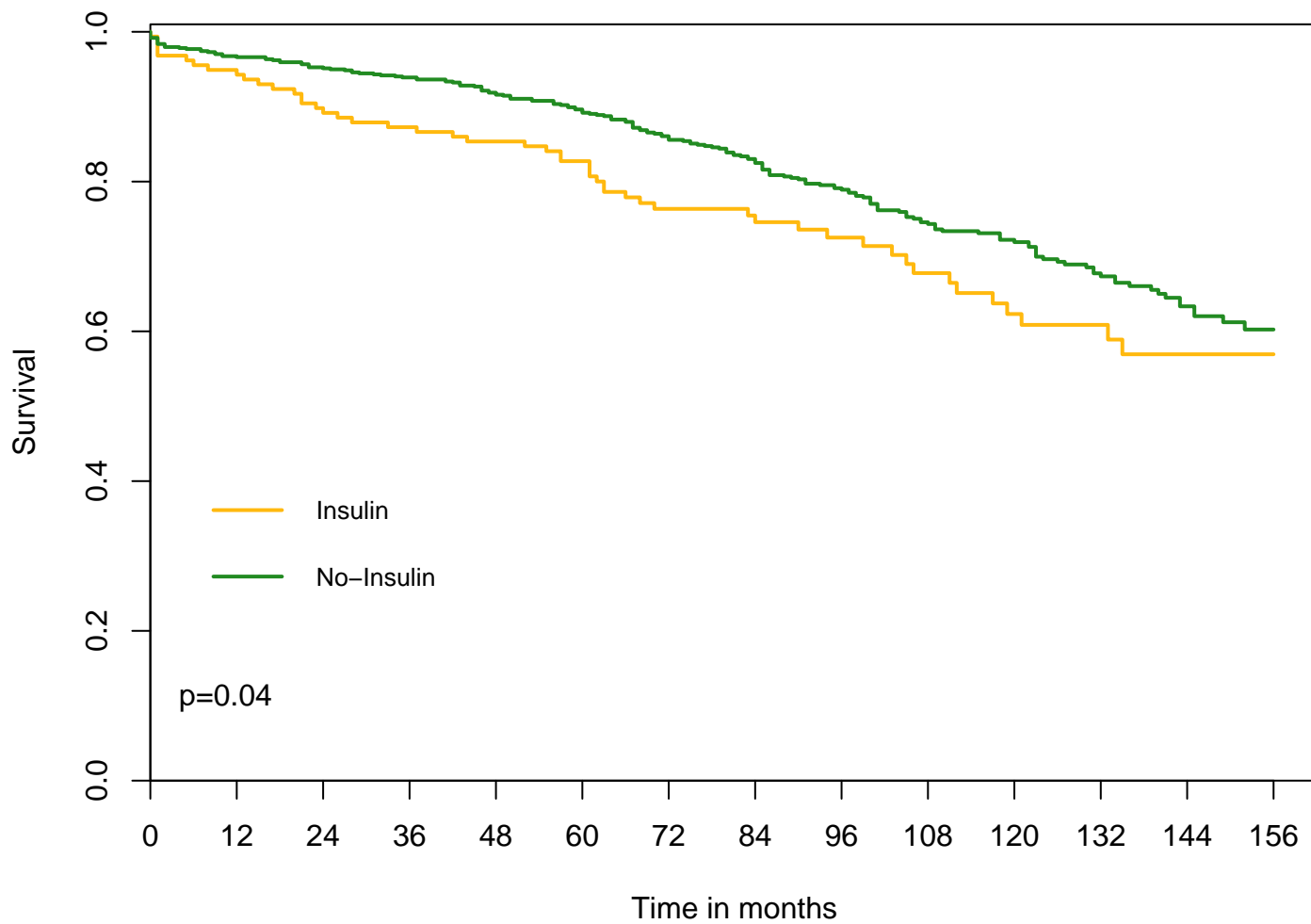

| # at risk  |     |                |     |     |     |     |     |     |     |     |     |     |     |     |     |
|------------|-----|----------------|-----|-----|-----|-----|-----|-----|-----|-----|-----|-----|-----|-----|-----|
|            |     | Time in months |     |     |     |     |     |     |     |     |     |     |     |     |     |
|            |     | 0              | 12  | 24  | 36  | 48  | 60  | 72  | 84  | 96  | 108 | 120 | 132 | 144 | 156 |
| Insulin    | 157 | 149            | 141 | 137 | 134 | 122 | 97  | 85  | 66  | 55  | 44  | 32  | 18  | 7   |     |
| No-Insulin | 738 | 714            | 703 | 693 | 677 | 608 | 527 | 468 | 391 | 319 | 235 | 167 | 106 | 41  |     |

Supplement: Supplementary file 4 — Additional file 4: Figure S3. Survival rate in the DM group receiving insulin treatment among patients with NYHA functional class I–II. DM = Diabetes mellitus; NYHA = New-York Heart Association. [file 12933_2018_796_MOESM4_ESM.pdf]

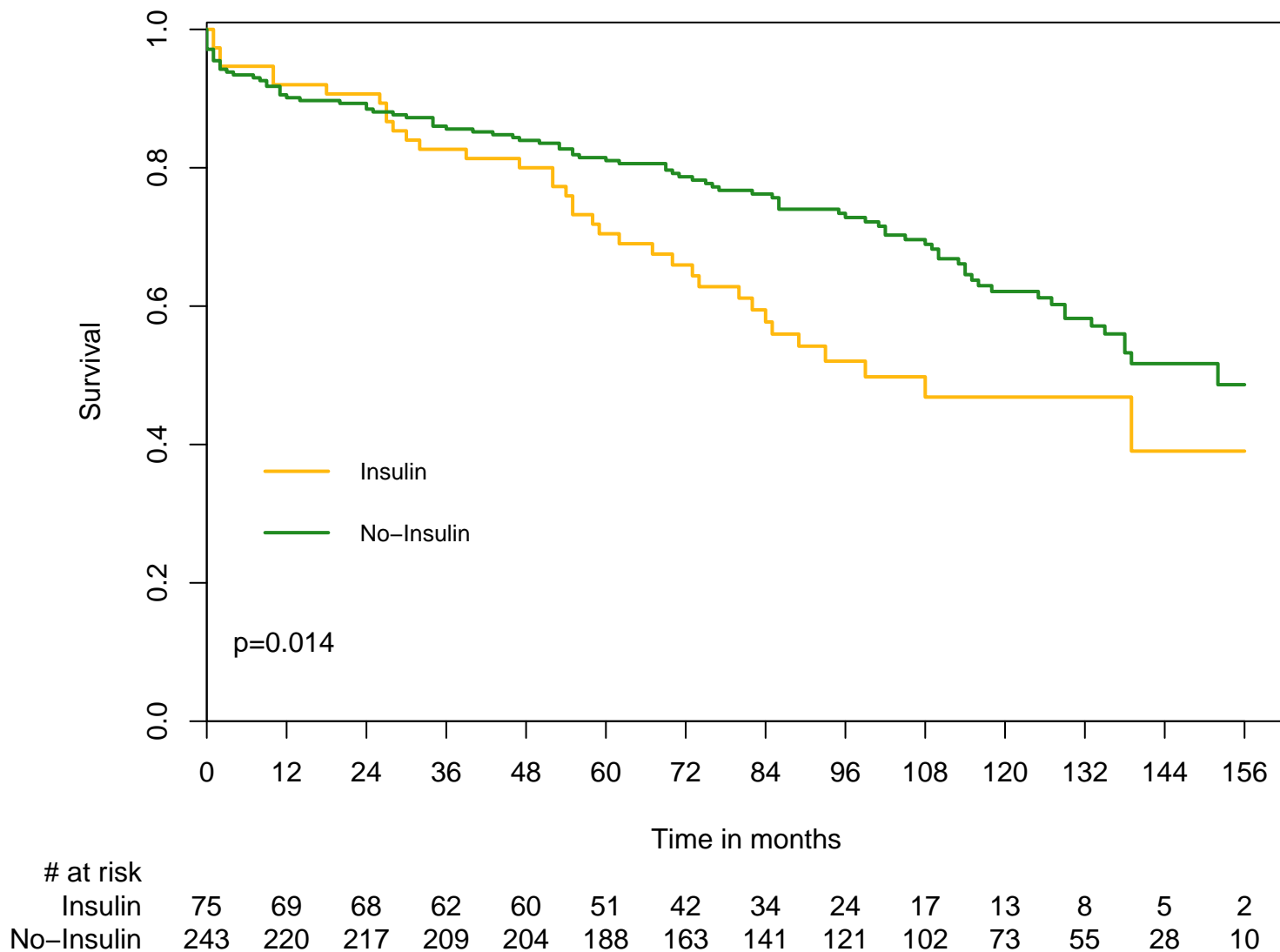

Supplement: Supplementary file 5 — Additional file 5: Figure S4. Survival rate in the DM group receiving insulin treatment among patients with NYHA functional class III–IV. DM = Diabetes mellitus; NYHA = New-York Heart Association. [file 12933_2018_796_MOESM5_ESM.pdf]
